# Structure of mycobacterial ergothioneine-biosynthesis C-S lyase EgtE

**DOI:** 10.1016/j.jbc.2023.105539

**Published:** 2023-12-10

**Authors:** Lili Wei, Lei Liu, Weimin Gong

**Affiliations:** School of Life Sciences, University of Science and Technology of China, Hefei, Anhui, China

**Keywords:** Mycobacterium, protein structure, redox homeostasis ergothioneine, ergothioneine-biosynthesis C-S lyase, MsEgtE, NcEgt2

## Abstract

L-ergothioneine is widely distributed among various microbes to regulate their physiology and pathogenicity within complex environments. One of the key steps in the ergothioneine-biosynthesis pathway, the C-S bond cleavage reaction, uses the pyridoxal 5′-phosphate dependent C-S lyase to produce the final product L-ergothioneine. Here, we present the crystallographic structure of the ergothioneine-biosynthesis C-S lyase EgtE from *Mycobacterium smegmatis* (MsEgtE) represents the first published structure of ergothioneine-biosynthesis C-S lyases in bacteria and shows the effects of active site residues on the enzymatic reaction. The MsEgtE and the previously reported ergothioneine-biosynthesis C-S lyase Egt2 from *Neurospora crassa* (NcEgt2) fold similarly. However, discrepancies arise in terms of substrate recognition, as observed through sequence and structure comparison of MsEgtE and NcEgt2. The structural-based sequence alignment of the ergothioneine-biosynthesis C-S lyase from fungi and bacteria shows clear distinctions among the recognized substrate residues, but Arg348 is critical and an extremely conserved residue for substrate recognition. The α14 helix is exclusively found in the bacteria EgtE, which represent the most significant difference between bacteria EgtE and fungi Egt2, possibly resulting from the convergent evolution of bacteria and fungi.

Tuberculosis, caused by *Mycobacterium tuberculosis* (Mtb), remains one of the deadliest infectious diseases in the world, and the co-infections of tuberculosis with COVID-19 and HIV have become a matter of global concern (1, 2). The Mtb is a well-equipped pathogen that poses a significant risk to human health, as it possesses inherent capabilities to adapt and reside within the phagosomes of human macrophage, persisting for several years, or even decades within specific granulomas (3). During the infection, Mtb is constantly exposed to oxidative stress. Therefore the homeostasis of oxidoreductive systems has a pivotal role in pathogenicity and survival of Mtb (4). Two major low-molecular-mass thiols synthesized by Mtb, mycothiol (MSH) and ergothioneine (EGT), maintain intracellular redox homeostasis (5, 6). Unlike MSH, EGT mainly exists in the form of thione rather than thiol at physiological pH, which makes it resistant to autoxidize (7).

EGT was first discovered in ergot fungi (8). Evidences have been provided that EGT with potent antioxidant properties protects cells from oxidative stress, owing to its 2-mercaptoimidazole side chain (9, 10). In mycobacteria, EGT plays a crucial role in maintaining redox and metabolic homeostasis to withstand various oxidative stressors and antibiotics, which is vital for the drug susceptibility and pathogenicity of Mtb (7, 9, 11). In addition, EGT has been reported to be applied in dietary supplements and cosmetic additives as well as for ameliorating various diseases (12, 13, 14) such as cardiovascular diseases (10, 15), neurodegenerative diseases (16), depression (17) and diabetes (18, 19). Human beings lack the homologous enzymes for the EGT biosynthesis and must ingest EGT from diet. Nevertheless, the extraction and purification of EGT from its natural sources is a complex undertaking, characterized by low yields and high production costs (20). Currently, the majority of EGT is manufactured through chemical synthesis (21), which is constrained by the safety concerns stemming from potential toxic by-products (22). Hence, the utilization of fungal or bacterial EGT biosynthesis pathways for the bioproduction of EGT at a low cost in a sustainable manner is increasingly garnering attention (12, 22, 23, 24).

Two typical EGT biosynthetic pathways have been identified previously (Fig. 1) (25). In the *Neurospora crassa* pathway, only two enzymes (Egt1 and Egt2) are involved in EGT biosynthesis (25, 26). While in the pathway of mycobacteria, a five-gene cluster (*egtABCDE*) is responsible for the biosynthesis of EGT, enabling mycobacteria to produce EGT from cysteine, glutamate, and histidine (25). EgtD is a histidine methyltransferase that catalyzes the trimethylated NH_2_ group of histidine to produce hercynine (TMH). Moreover, EgtA catalyzes the synthesis of γ-glutamylcysteine (γ-GC). Subsequently, in the presence of oxygen and FeSO4, EgtB catalyzes the formation of S-hercynyl-γ-glutamylcysteine sulfoxide from γ-GC and TMH. The glutamine amidotransferase EgtC eliminates the glutamine fraction to generate sulfoxide 4, which is finally cleaved by the PLP-dependent C-S lyase EgtE to produce EGT.

**Figure 1:**
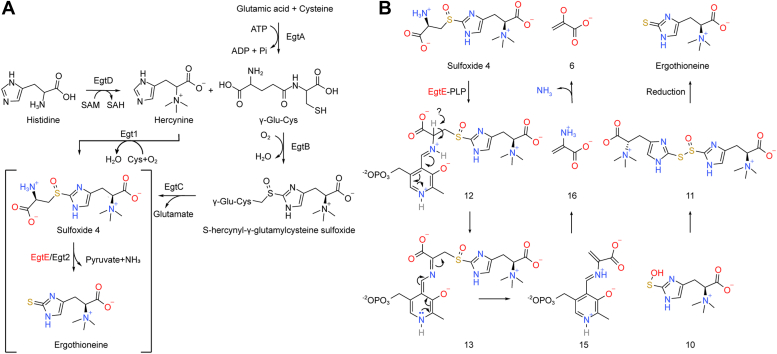
**Ergothioneine biosynthesis pathways and the catalytic mechanism of EgtE.***A*, pathways involved in ergothioneine biosynthesis are found in mycobacteria (EgtA-E) and *N. crassa* (Egt1 and Egt2). *B*, catalytic mechanism of EgtE in mycobacteria, as previously reported.

The pathway of EGT biosynthesis may be a potential drug target for the treatment of tuberculosis (9). The inhibitors targeting enzymes involved in EGT biosynthesis have been reported. Buthionine sulfoximine (BSO), a γ-glutamylcysteine synthetase inhibitor that inhibits eukaryotic GSH synthesis, has been shown to strongly repress the activity of EgtA (27, 28). Besides, the structures of EgtB, EgtC, and EgtD are unveiled, showing the framework of their active sites (29, 30). Moreover, the structures of EgtD complex with inhibitors suggest that EgtD is a feasible target for a novel therapy against multidrug-resistant Mtb (28, 31).

C-S bond formation and breakage are the key steps in EGT biosynthesis (32). EgtE and Egt2 are PLP-dependent C-S lyases in EGT biosynthesis, leading to the conversion from sulfoxide 4 to EGT. Due to the difficulties in crystallization, the structure of EgtE from mycobacteria has not been resolved yet, while the crystal structure of Egt2 in the *N. crassa* pathway (NcEgt2) has been published (32).

In this study, we report the crystal structures of EgtE from *Mycobacterium smegmatis* (MsEgtE) and identify several key residues involved in substrate binding and catalysis. The role of these residues was confirmed by site-directed mutagenesis. The structural comparison between MsEgtE and NcEgt2 reveals distinct substrate recognition models in the two different EGT biosynthetic pathways. Through sequence and structural conservation analyses of the EGT-biosynthesis C-S lyases, we identify the most significant structural difference (α14 helix) between fungal Egt2 and bacterial EgtE and the decisive residue (Arg348) determining substrate specificity. The structural basis of MsEgtE further validates the presence of intermediate in the catalytic process and provides new ideas for the development of anti-tuberculosis drugs targeting EGT biosynthesis pathway and the optimization of recombinant microbial fermentation to produce EGT (9, 20, 33, 34).

## Results

### Overall structure of MsEgtE

Although full-length EgtE from *M. smegmatis* (MsEgtE) is highly challenging to crystallize (32), we have successfully obtained crystals of MsEgtE sharing 65.75% sequence identity with *M. tuberculosis* EgtE (MtEgtE) (Fig. S1*A*). The structure of MsEgtE was solved by molecular replacement using the model of NcEgt2 (PDB code: 5UTS) from *N. crassa*, showing less than 20% amino acid sequence similarity with MsEgtE.

**Figure 2:**
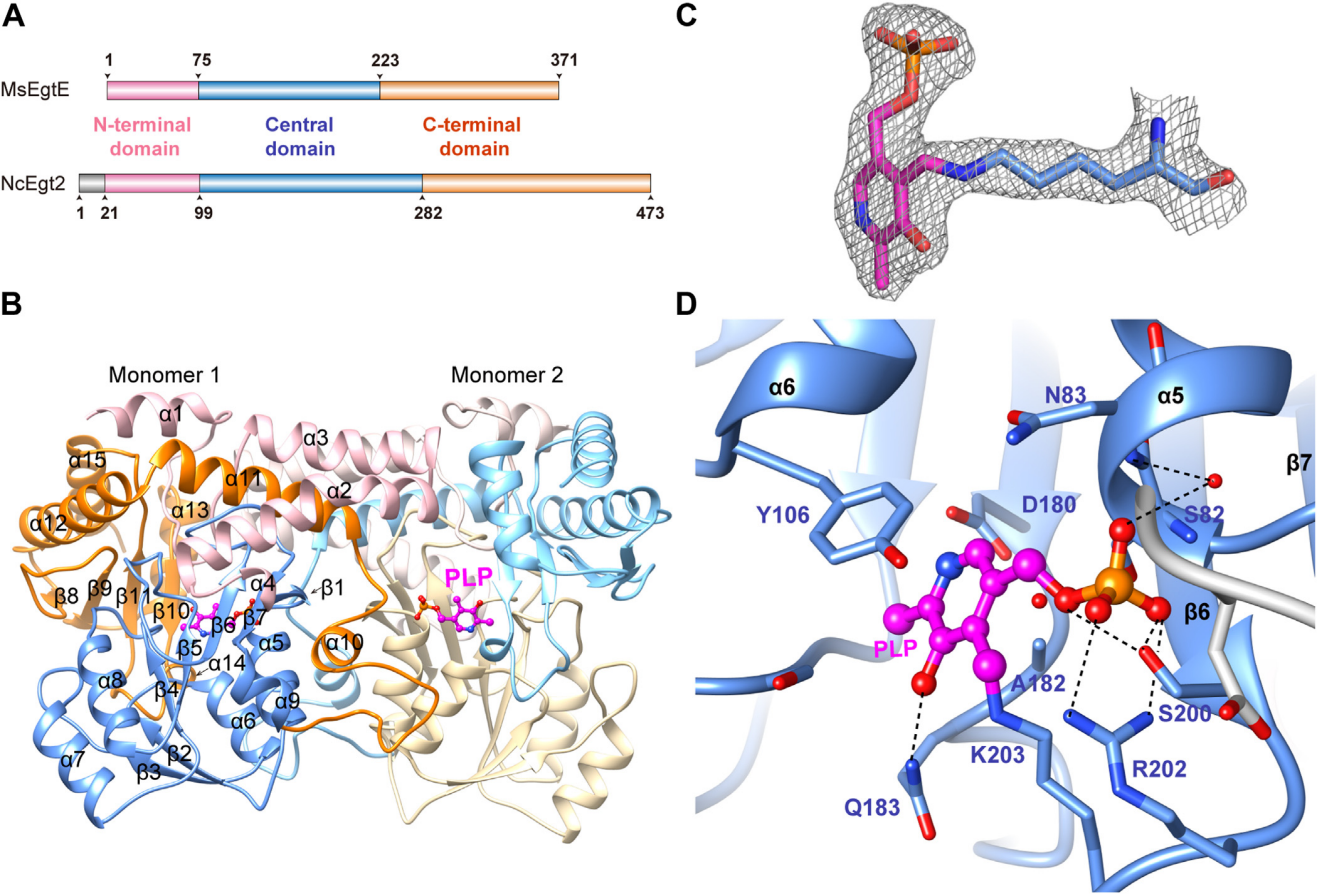
**Overall structure of MsEgtE and structure of MsEgtE-PLP.***A*, based on their monomer structures, MsEgtE and NcEgt2 are divided into three domains. The N-terminal domain is shown in *pink*, the central domain is shown in *blue*, and the C-terminal domain is shown in *orange*. There are not any homologous amino acids in MsEgtE that correspond to the *gray* sequence in NcEgt2. *B*, ribbon representation of MsEgtE dimer. The PLP cofactors are represented as *balls and sticks*. The N-terminal domain, central domain, and C-terminal domain from monomer 1 are distinguished with *pink, blue*, and *orange colors*, respectively. And the three domains from monomer 2 are distinguished with *light pink*, *wheat*, and *sky blue*, respectively. *C*, electron density map of PLP covalently binding to Lys203 of the MsEgtE-PLP complex. The 2Fo-Fc electron density maps of PLP and Lys203 are shown with a *gray*-colored mesh and contoured at 1.5σ. *D*, residues interacting with PLP are represented as *sticks*, and hydrogen bonds formed with PLP in the MsEgtE-PLP complex are shown as *black dashed lines*. *Red* dissociative *balls* represent water molecules.

The overall structure of MsEgtE was determined with a resolution of 2.8 Å and in good agreement with the X-ray crystallographic statistics for bond angles, bond lengths, and other geometric parameters (Table S1), which exhibits two molecules per asymmetric unit. Each MsEgtE monomer has 15 α-helices and 11 β-strands and is classified into three domains based on the domain assignments of NcEgt2 (Fig. 2*A*). Structurally, the N-terminal domain of MsEgtE contains residues 1 to 75 forming four helices (α1-4). The large central domain contains residues 76 to 223 generating a typical α/β fold (Fig. 2*B*), which is consistent with the conserved folding in type 1 PLP-dependent enzymes (aspartate aminotransferase family) (35). In the central domain, MsEgtE has seven β-strands (β1-7) parallel to each other except the antiparallel strand of β7 and surrounded by five α-helices, but has no helix connection between β-strands 5, 6, and 7 (Fig. 2*B*). The C-terminal domain (residues 224–371) consists of six α-helices (α5-9) and four-stranded antiparallel β-sheets (β8-11) (Fig. 2*B*). Dimerization of MsEgtE in the crystal is consistent with size exclusion chromatography measurements of molecular masses with a value of ∼76 kDa, close to the predicted value of 78 kDa expected for a dimer.

### Active site of MsEgtE

The PLP cofactor is buried within the conserved active site of MsEgtE (Fig. 2*B*). The PLP-binding pocket is mainly composed of residues from the central domain and a loop from the neighboring monomer. A total of 60 amino acids from each monomer are involved in dimerization, which are mainly mediated through the contact between seven α-helices (α2, α3, α5, α6, α11, α13, α14) and five loops (residues 12–27, 202–211, 227–230, 246–250, 329–331) of one monomer and the corresponding structures of the other monomer (Fig. 2*B*). Approximately 13% of the monomer surface contributes to dimer formation.

PLP covalently linked to Lys203 *via* a Schiff base linkage in monomer 1, with NZ-C4′ distance of 1.3 Å. The O3′ atom of PLP forms a hydrogen bond with Gln183 (Fig. 2*D*). The pyridine ring of PLP is sandwiched between Tyr106 and Ala182, which shows that the aromatic ring of Tyr106 and the sidechain of Ala182 provide hydrophobic interactions at the two sides of the pyridine (Fig. 2*D*). To strengthen the orientation, a salt bridge forms between the nitrogen of pyridine and Asp180. Besides, another salt bridge between the phosphate group of PLP and Arg202 also anchors the position of the PLP cofactor (Fig. 2*D*). In addition, the phosphate group of PLP is tightly bound *via* hydrogen bonds with Ser82, Asn83, and Ser200, which forms an extensive network of hydrophilic interactions to further limit the movements of the cofactor (Fig. 2*D*).

Conversely, in the monomer 2, the Schiff base bond is broken, resulting in the increased NZ-C4′ distance of 3.0 Å (Fig. S2). The lack of a covalent bond between lysine and PLP has also been observed in other PLP-dependent enzymes, believed to be caused by radiation-induced damage during the data collection process (36, 37). In order to investigate whether this was induced by radiation damage in MsEgtE, the structure was refined against the early or late halves, respectively. However, no significant differences were observed in PLP linkage (Fig. S2). Therefore, whether radiation damage occurred in this case needs further investigation.

### Structure of MsEgtE-PLP bound with pyruvate or intermediate

**Figure 3:**
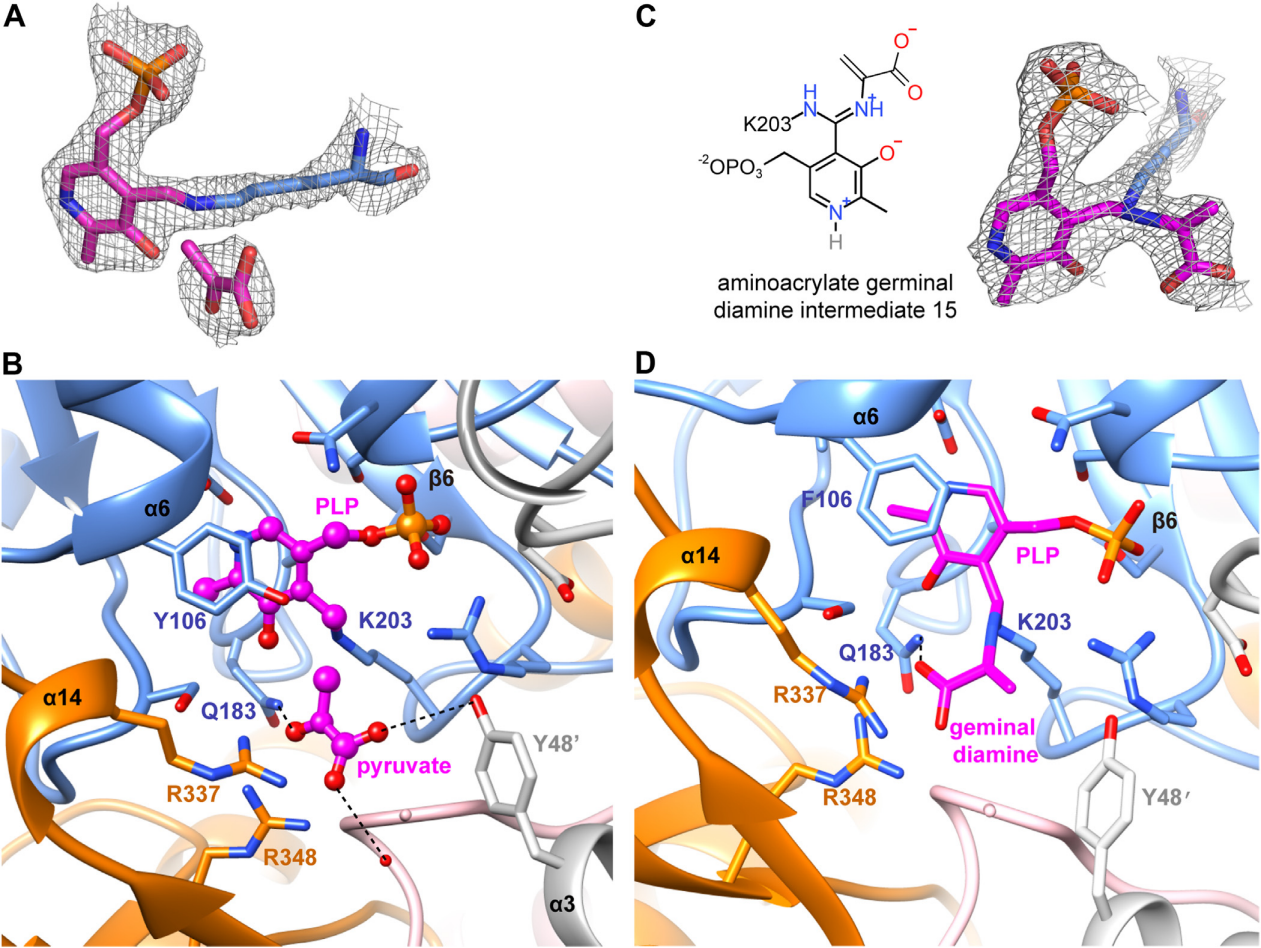
**Structures of MsEgtE-PLP binding pyruvate and MsEgtE(Y106F)-PLP-geminal diamine complex.***A*, pyruvate and PLP in the MsEgtE active site are shown with 2Fo-Fc electron density maps (*gray*-colored mesh, 1.5σ). *B*, interaction network of pyruvate with MsEgtE and PLP. Hydrogen bonds formed with pyruvate are shown as *black dashed lines*. *C*, geminal diamine and PLP covalently binding to Lys203 in the MsEgtE(Y106F) active site are shown with 2Fo-Fc electron density maps (*gray*-colored mesh,1.5σ). *D*, interaction network of geminal diamine with MsEgtE(Y106F) and PLP. Hydrogen bonds formed with geminal diamine are shown as *black dashed lines*.

To explore the catalytic mechanism of EGT formation from sulfoxide 4, we further determined the MsEgtE structures with substrate or intermediates. Because of the fast catalytic turnover of the enzyme in the substrate as described in NcEgt2 (32), it is hard to obtain MsEgtE–substrate complex by co-crystallization. A data set to 2.35 Å resolution was collected from a MsEgtE crystal with a short soak in the substrate (Table S1). We observed an additional oval-shaped electron density in the active site, but it does not match the substrate (sulfoxide 4). Based on the catalytic mechanism of the NcEgt2 enzyme, we found that pyruvate, one of the products in the catalytic reaction, fits well into the electron density map (Figs. 1*B* and 3*A*). The complex of MsEgtE-PLP binding to pyruvate is constructed by a homotetramer composed of two homodimers with two PLP cofactors located close to their respective subunits, and pyruvate interacted with the interface of the A/C subunit (Fig. 3*B*).

In the structure of the MsEgtE-PLP binding pyruvate complex, pyruvate binds in the deep crevice of the active center *via* extensive interaction with surrounding residues. Specifically, pyruvate forms salt bridges with Arg348 and Arg337, and also binds to Gln183 through a hydrogen bond (Fig. 3*B*). Besides, the carboxyl group of pyruvate creates hydrogen bonds with Tyr48′ of another monomer and water molecule (Fig. 3*B*).

In addition, we also found the electron density of aminoacrylate geminal diamine intermediate 15 in the structure of MsEgtE(Y106F)-PLP-geminal diamine complex (Fig. 3*C*). The geminal diamine and PLP C4′ are both covalently linked to the ε-amino of Lys203 (Fig. 3*D*). The captured structure confirms the presence of the intermediate 15, which is formed by transaldimination with Lys203 during the C-S cleavage reaction. This reaction ultimately results in the formation of pyruvate and ammonia, while simultaneously regenerating Lys203.

### MsEgtE activity and kinetic characterization

The structures of the MsEgtE described above shed light on several residues (Tyr48, Tyr106, Asp180, Gln183, Arg202, Lys203, and Arg348) in the active pocket, which potentially contribute to substrate recognition and catalysis. To understand the role of these residues in MsEgtE mediated reaction, site-directed mutagenesis experiments were performed to yield mutants of Y48A, Y106F, D180A, Q183A, R202A, R202K, K203A, and R348A (Fig. S7). These mutants all retain varying degrees of PLP binding affinity (Fig. S3*A*). The kinetic constants of the wild-type and mutants were then determined using pyruvate and lactate dehydrogenase coupling assay.

In PLP-dependent enzymes, PLP serves as an electron sink, promoting the resonance stabilization of electrons or negative charges (38). Maintaining the immobility of PLP during catalysis is crucial for efficient enzyme activity.

**Figure 4:**
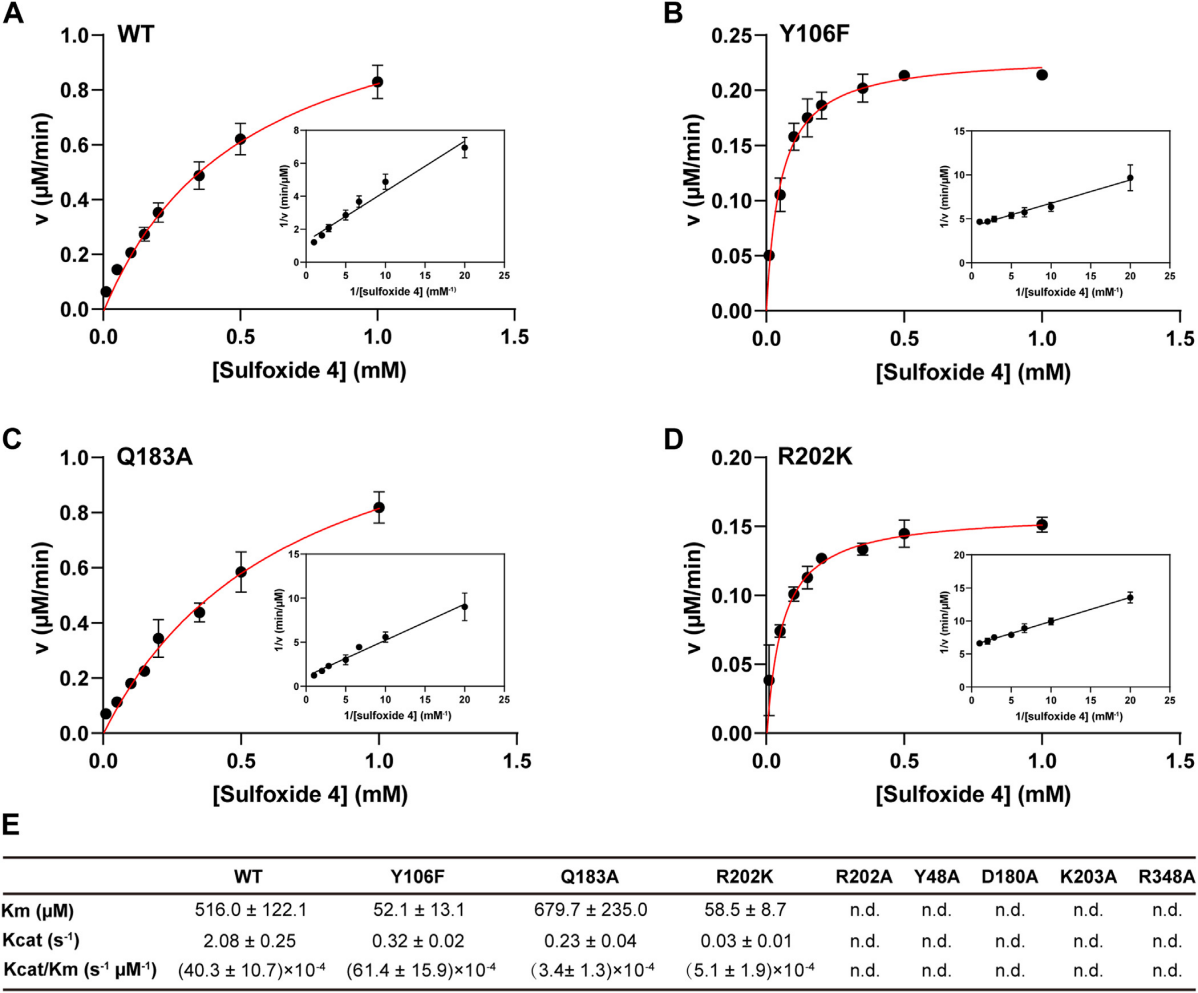
**Steady-statekinetic analysis of MsEgtE wild-type and mutants at 25 °C.***A*–*E*, The steady-state kinetic analysis of wild-type MsEgtE (WT) and mutants was determined using a coupled assay by monitoring absorbance at 340 nm. Results are shown as means ± SD (n = 4). The data were fitted by MATLAB 2022b.

The covalent linkage between Lys203 and PLP through the Schiff base is conserved in PLP-dependent enzymes and plays a vital role in PLP binding and substrate catalysis (39, 40). It facilitates the formation of external aldimines and enzyme-substrate intermediates, as well as the release of products (41, 42). Asp180 forms a salt bridge with the pyridine nitrogen of PLP, governing the electron sink properties of PLP and stabilizing the protonated state of PLP N1 protons (43), which is conserved in type 1 PLP-dependent enzymes. As expected, both K203A and D180A lose enzyme activity, although they still retain some ability to bind PLP (Figs. 4*E* and S3*A*).

Gln183 is involved in both PLP binding and pyruvate generation (Fig. 3*B*). Mutating Gln183 to alanine does not affect PLP binding, but the *k*_cat_/*K*_m_ value is reduced by 11.8-fold less than that of wild type (Figs. 4, *C* and *E*, and S3*A*). This indicates that Gln183 exerts a greater influence in the substrate catalysis compared to its role in the PLP binding. In MsEgtE, the phosphate group of PLP is stabilized by salt bridges with Arg202 and a network of hydrogen bonds contributed by neutral amino acid residues and water molecules (Fig. 2*D*). The R202K mutant results in a 7.9-fold decrease in *k*_cat_/*K*_m_ compared to the wild type (Fig. 4, *D* and *E*). Additionally, both R202A and R202K mutations lead to almost complete loss of catalytic activity, highlighting the importance of phosphate group fixation for the catalytic process (Fig. 4, *D* and *E*). Remarkably, phenylalanine substitution for Tyr106 results in a 6.5-fold decrease in *k*_cat_, while a 1.5-fold increase in catalytic efficiency (*k*_cat_/*K*_m_) compared with the wild type (Fig. 4, *B* and *E*). This implies that Y106 plays a crucial role in substrate binding, and specifically, the phenolic hydroxyl group of Y106 contributes to the substrate catalysis.

In PLP-dependent enzymes, Arg is responsible for recognizing the carboxylate substrate, as well as stabilizing substrate binding and intermediate resonance states (37, 38, 44). The R348A mutant is functionally inactive, providing conclusive evidence that Arg348 plays a critical role in both substrate carboxyl group recognition and catalysis (Figs. 4*E* and S3*B*). Notably, the Y48A mutant exhibits thermodynamic change upon substrate interaction in isothermal titration calorimetry (ITC) experiments, despite its inability to generate pyruvate (Figs. 4*E* and S3*B*), which indicates the Y48A mutant still retains a certain level of substrate binding capability.

### Sequence alignment and structural analysis of MsEgtE and NcEgt2

**Figure 5:**
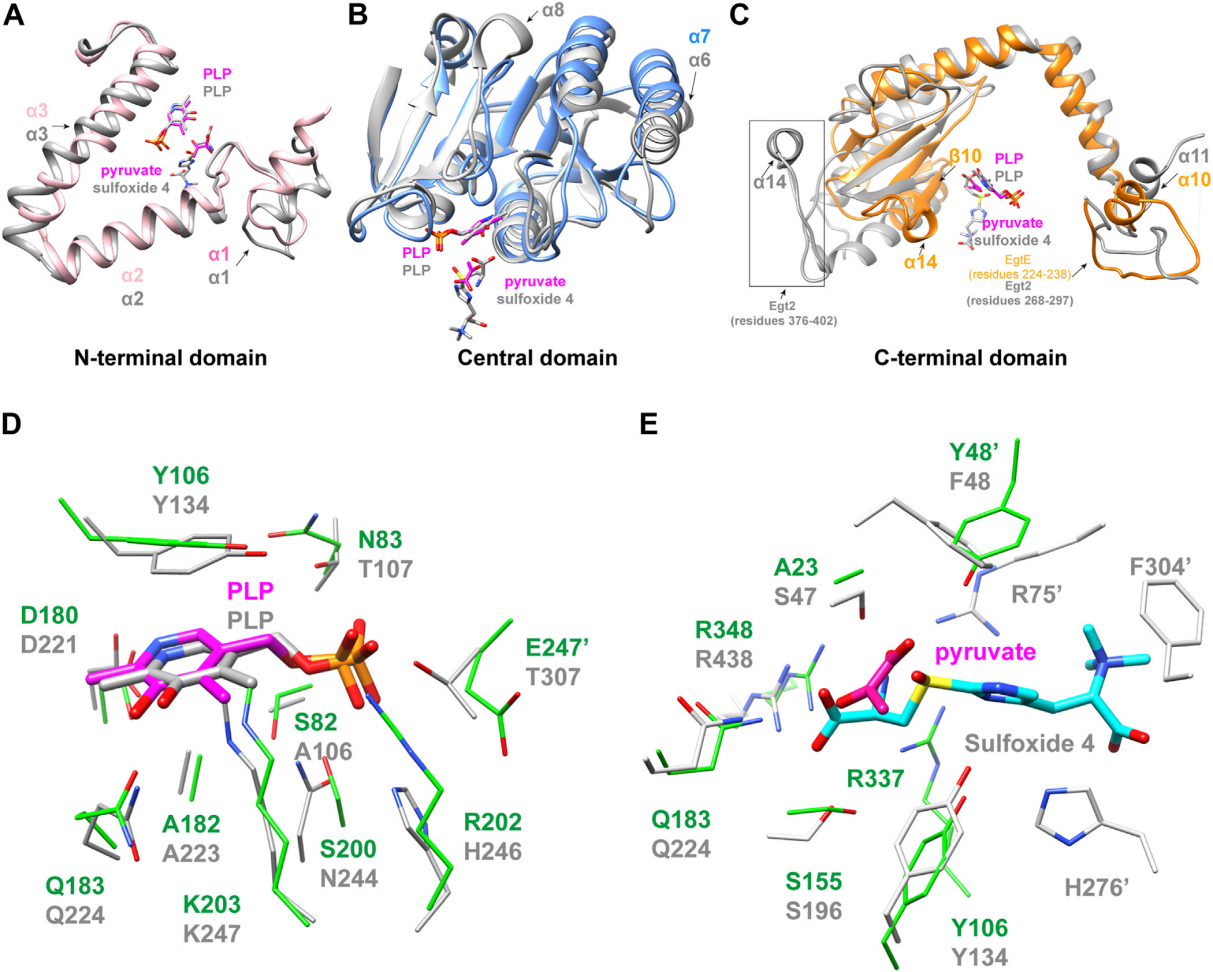
**Structural alignment of MsEgtE and NcEgt2.***A–C*, the structural alignments of different domains in MsEgtE-PLP binding pyruvate and NcEgt2-PLP binding sulfoxide 4 complexes. MsEgtE (The N-terminal domain, central domain, and C-terminal domain are distinguished with *pink*, *blue*, and *orange colors*, respectively.) and NcEgt2 (*dark gray*) are ribbon representations. PLP (colored *magenta* in MsEgtE and colored *dark gray* in NcEgt2), pyruvate (*magenta*), and sulfoxide 4 (*dark gray*) are represented as *sticks*. *D*, comparison of PLP (*magenta* from MsEgtE, *dark gray* from NcEgt2) and its interacting amino acids in MsEgtE (*green*) and NcEgt2 (*dark gray*) active sites respectively. *E*, comparison of pyruvate (*magenta*)/sulfoxide 4 (*dark gray*) and their interacting amino acids in MsEgtE (*green*)/NcEgt2 (*dark gray*) active sites. Residues with superscript markers mean these residues from the neighboring monomer.

In the EGT biosynthetic pathway, both MsEgtE and NcEgt2 act as key enzymes in the PLP-catalyzed C-S lyase reaction that transfers a sulfur atom of sulfoxide 4 from cysteine to histidine side chain (32). Besides, their quaternary structures also exhibit a significant degree of similarity (RMSD = 2.53 Å). Despite their functional and structural similarity, they exhibit low amino acid sequence homology and some variations in the active sites (Fig. 5). In type 1 PLP-dependent enzymes, the N- and C-terminal domains exhibit significant diversity, suggesting that their functions primarily associated with substrate specificity or regulation (32). In comparison to MsEgtE, NcEgt2 has an additional sequence preceding the N-terminal domain and a more variable C-terminal domain, while their N-terminal and central domains remain relatively conserved (Figs. 2*A* and 5, *A*–*C*).

In the N-terminal domains of MsEgtE and NcEgt2, α2 and α3 are consistent, but α1 is slightly different (Fig. 5*A*). α2 and α3 are inserted into the active center of the neighboring monomer, with α3 directly participating in substrate binding (Figs. 3*B* and 5). The large central domains of the two enzymes both display a typical α/β folding, which is conserved in type 1 PLP-dependent enzymes. However, there are slight differences in helical length and spatial position between α7 of MsEgtE and α6 of NcEgt2, and NcEgt2 also has an additional distinctive structure, α8 (Fig. 5*B*). Nevertheless, these helices are not involved in substrate binding and catalysis.

**Figure 6:**
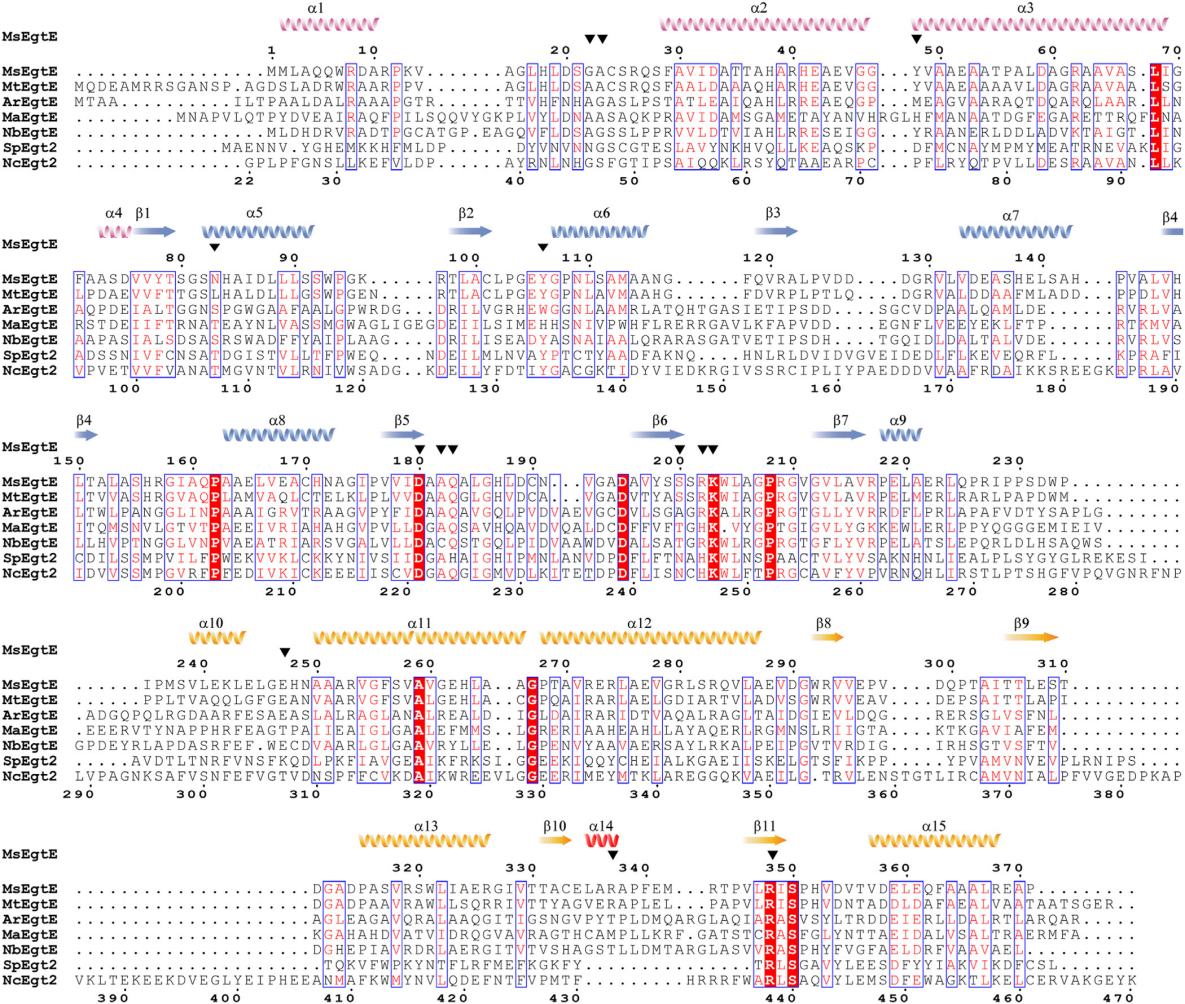
**Structure-based sequence alignment of ergothioneine-biosynthesis C-S lyases from different species.** Species abbreviations: Ms, *Mycolicibacterium smegmatis*; Mt, *Mycobacterium tuberculosis*; Ar, *Achromobacter ruhlandii*; Ma, *Methylobacterium aquaticum*; Nb, *Nocardia brasiliensis*; Sp, *Schizosaccharomyces pombe*; Nc, *Neurospora crassa*. The structure of MtEgtE, ArEgtE, MaEgtE, NbEgtE, and SpEgtE have been predicted by AlphaFold. The secondary structural elements of MsEgtE are mapped along its sequence. α helixes are represented by the spiral coils and β sheets are represented by arrows. Different colors correspond to the three domains of MsEgtE. α14 is the most distinct structure in bacteria and fungi and is represented by a *red spiral coil*. The active site residues MsEgtE are represented by the *black*.

The C-terminal domain of MsEgtE is significantly smaller than that of NcEgt2. Although both the loop (residues 224–238) of MsEgtE and the loop (residues 268–297) of NcEgt2 are located at the interaction interface of their dimers (Fig. 5*C*), only the loop (residues 268–297) of NcEgt2 directly interacts with the histidine portion of the substrate (32). The α14 in the C-terminal domain from MsEgtE is a unique secondary structure that is absent in NcEgt2 (Figs. 5*C* and 6). This α14 occupies a specific space in the substrate binding pocket, generating specific interactions that result in a tighter binding of the cysteine portion of the substrate by MsEgtE compared to NcEgt2. Additionally, α14 (residues 390–396) from NcEgt2 and two long loops at its ends (residues 376–402) are also unique secondary structures (Figs. 5*C* and 6). However, these structures are located far from the active site and unrelated to dimer formation, suggesting that they may be redundant structures.

### Active center comparative analysis of MsEgtE and NcEgt2

The active sites of MsEgtE and NcEgt2 primarily consist of the central domain and C-terminal domain, with a partial contribution from the N-terminal and C-terminal domains of the neighboring monomer. Residues binding with the cofactor PLP are conserved in MsEgtE and NcEgt2. PLP is covalently linked to Lys203/247 (number in MsEgtE/NcEgt2) and anchored by π-π stacking interactions with Tyr106/134 which exhibit different orientations of the side-chain aromatic ring (Fig. 5*D*). The pyridine ring of PLP is also stabilized by salt bridges with Asp180/221 and hydrogen bonds with Gln183/224. However, the amino acids (Ser82/Ala106, Asn83/Thr107, Ser200/Asn244, Arg202/His246) involved in interactions with the phosphate group of PLP show less conservation. Moreover, Glu247 in MsEgtE, a negatively charged residue from the neighboring monomer, interacts with water molecular that engages in hydrogen bond with the PLP phosphate group, while in NcEgt2, Thr307 directly forms hydrogen bonds with the phosphate group (Fig. 5*D*).

In the substrate binding sites of MsEgtE and NcEgt2, only residues (Arg348/438, Gln183/224, Ser155/196, Tyr106/134) are conserved (Fig. 5*E*). In the α14 of MsEgtE, Arg337 directly interacts with the carboxyl groups of the substrate's cysteine part and forms salt bridges (Fig. 5*E*). However, NcEgt2 lacks a corresponding structure and residues similar to the α14 position of MsEgtE. Tyr48 on α3 of MsEgtE, occupying a spatial position similar to the Phe48 in NcEgt2, participates in pyruvate production with the same function as Arg75 from α3 in NcEgt2 (Fig. 5*E*). Additionally, Phe48, Phe304 and His276 of NcEgt2 are involved in binding the histidine part of the substrate, but these residues have no homologous amino acids in MsEgtE (Fig. 5*E*). In brief, MsEgtE binds more tightly to the cysteine portion but much more loosely to the histidine portion of the substrate compared to NcEgt2.

In the mechanistic model for NcEgt2 catalysis, the formation of a disulfide bond between the ergothioneine sulfonic acid intermediate (10) and C156 at the exit of the active center is described (32). This disulfide bond is subsequently reduced by a reductant, leading to the release of EGT from the active center and the restoration of C156 for the next catalytic cycle. However, in the case of MsEgtE, the structure reveals the absence of a homologous residue to C156, and the exit of its active center is obscured by α14, β10, and several loops. To investigate whether a similar mechanism is at play in the catalytic process of MsEgtE, we conducted a single-turnover experiment. The goal was to determine whether the intermediate (10) could form covalent adducts with the cysteine residues in MsEgtE. Equal molar ratios of protein and substrate were incubated, followed by trypsin digestion of the protein in the absence of a reductant. Unreacted protein was included as a control. The LC-MS/MS analysis revealed the presence of covalently modified sulfonic acid adducts on C24, C101, and C190. However, the XCorr values obtained for these adducts (0.74, 0.97, and 1.1, respectively) suggest that these results are not reliable. Based on these findings, we speculate that the formation of a disulfide bond between the intermediate (10) and cysteine residues is not a necessary step in the catalytic reaction. Instead, it may be a consequence of the instability of the intermediate (10). This unstable intermediate could either undergo a disproportionation reaction, resulting in the formation of a sulfonate (11) or bind to cysteine residues to form a disulfide bond.

## Discussion

In this study, we present the crystal structure of EgtE from *Mycobacterium smegmatis* which represents the first published structure of EGT-biosynthesis C-S lyases in bacteria. The structure of MsEgtE further corroborates and complements the previously hypothesized catalytic mechanism of EgtE, and confirms the existence of aminoacrylate geminal diamine intermediate 15 during the catalytic process (Figs. 1*B* and S6). Notably, a structural superposition of MsEgtE with NcEgt2 as the reference reveals distinct differences in substrate binding modes and a low sequence similarity between them, despite their overall structural similarities. Compared to NcEgt2, the presence of α14 in the structure of MsEgtE leads to a stronger binding between MsEgtE and the cysteine moiety of the substrate. Conversely, the binding between MsEgtE and the histidine moiety is relatively weaker. Therefore, it is believed that focusing on the design of a C-S cleavage enzyme with high affinity for sulfone 4, centered around EgtE α14, may be an effective approach to enhance the yield of EGT biological fermentation.

Furthermore, we conducted phylogenetic tree analysis using ClustalW and MEGA-X to investigate the relationship between MsEgtE and other EGT-biosynthesis C-S lyases. Our analysis focuses on EGT-biosynthesis C-S lyases from fungi and bacteria (45, 46), and predicts the structures of EGT biosynthetic C-S lyases from different species (Figs. S4 and S5). The structure-based sequence alignment of EGT-biosynthesis C-S lyases shows that the PLP-binding residues are essentially conserved, whereas the substrate-binding residues, with the exception of Arg348, display a lack of conservation (Fig. 6). According to the aforementioned results, we can conclude that the Arg348 is the crucial residue that determines the substrate specificity of the EGT-biosynthesis C-S lyases. Interestingly, the presence of α14 in MsEgtE is exclusive to bacteria, particularly actinomycetes and proteobacteria, while fungi lack this characteristic (Fig. S5). This observation implies that fungal and bacterial EGT-biosynthesis C-S lyases may have independently evolved to catalyze the same reaction in different environments, resulting in similar active sites and overall structures.

It is worth mentioning that the predicted structure of *M. tuberculosis* is remarkably similar to the monomer structure of EgtE from *Mycobacterium smegmatis*, consistent with their highly conserved sequences (Fig. S1). And the residues in the active centers of MsEgtE and MtEgtE exhibit nearly identical PLP binding and substrate recognition (Fig. S1, *B* and *C*). Given the high conservation of structures and residues involved in catalysis in mycobacterial EgtE, we believe that drugs designed to target the active site of EgtE based on the structure of MsEgtE may directly inhibit the mycobacterial EGT biosynthesis pathway and potentially impede the development of drug resistance in *M. tuberculosis*.

## Experimental procedures

### Reagent

The screening solutions used for the experiments are from Hampton. All crystallization reagents are obtained from Sigma-Aldrich. EGT was purchased from Santa Cruz Biotechnology. Sulfoxide 4 was provided by Pinghua Liu’s laboratory.

### Cloning, expression, and purification of proteins

The full-length ORF of *msmeg_6246* [*egtE* gene; National Center for Biotechnology Information (NCBI) gene ID: 4531386; UniProt accession no. A0R5M7] was PCR-amplified from the *M. smegmatis* strain mc^2^155 genomic DNA library and subcloned into the expression vector pET28a containing a N-terminal hexa-histidine tag. Mutations were generated by site-directed mutagenesis. All primers used in molecular cloning and mutagenesis were listed in Table S2 and all fusion constructs are expressed in *Escherichia coli* BL21 (DE3) cells (Novagen) in LB medium supplemented with 30 μg/ml kanamycin.

Recombinant proteins were expressed and purified as previously reported (47, 48). The cells were grown at 37 °C to an OD_600_ of 0.8 and then induced with 0.2 mM IPTG (isopropyl-β-D-1-thiogalactopyranoside; Sigma-Aldrich) for 20 h at 16 °C.

Subsequently, the cells were harvested by centrifugation at 4000 rpm for 30 min at 4 °C, and cell pellets were resuspended in buffer A (20 mM Tris-HCl, pH 8.5, 1 M NaCl, 0.5 mM PLP, 1 mM PMSF). Bacterial cells were lysed by homogenization, and the lysate is centrifuged at 16,000 rpm for 30 min at 4 °C. The supernatant was purified by Ni-affinity chromatography (Chelating SepharoseTM Fast Flow, GE Healthcare) equilibrated with buffer B (20 mM Tris-HCl, pH 8.5, 1 M NaCl). Then, the beads were washed with buffer C (20 mM Tris-HCl, pH 8.5, 1 M NaCl, 40 mM imidazole) followed by the elution with buffer D (20 mM Tris-HCl, pH 8.5, 1 M NaCl, 300 mM imidazole). All proteins from the eluted fraction were further purified on a HiLoad 16/600 Superdex 200 pg (GE Healthcare) in buffer B at 4 °C. After purification, the protein concentration is determined by measuring the absorbance at 280 nm using a theoretical extinction coefficient for the protein. Mutant proteins were prepared using the same protocol described above.

### Crystallization

Crystallization of MsEgtE-PLP was performed in sitting drop vapor diffusion plates at 16 °C (49, 50). Crystals of the MsEgtE-PLP grow in 0.1 M HEPES-Na, pH 7.5, 1 M NaCl, 0.1 M LiSO4, 10% PEG1000. Glycerol (40%, v/v), and 20 mM substrate were used as a cryoprotectant before being obtained and frozen in liquid nitrogen.

Crystals of MsEgtE-PLP-pyruvate complex were achieved by mixing MsEgtE-PLP [incubated with 5 mM EGT (SCBT, SC201814)] with 1 μl reservoir solution equilibrated against precipitating solution (0.1 M citric acid, pH 6.5, 15% PEG8000, 15% glycol), and crystals were cryoprotected in reservoir solution supplemented with glycerol (40%, v/v) and sulfoxide 4 (10 mM) before being frozen in liquid nitrogen.

Crystals of MsEgtE(Y106F)-PLP-geminal diamine complex are achieved by mixing MsEgtE(Y106F) [incubated with EGT (5 mM)] with 1 μl reservoir solution equilibrated against precipitating solution (0.1 M HEPES-Na, pH 7.5, 12% PEG20000), and crystals are cryoprotected in reservoir solution supplemented with glycerol (40%, v/v) and sulfoxide 4 (10 mM) before being frozen in liquid nitrogen.

### Data collection, structure determination, and refinement

All diffraction datasets were collected at beamline BL17U at the Shanghai Synchrotron Radiation Facility (SSRF). For the MsEgtE-PLP crystal, 361 frames were collected with an oscillation step of 1° per frame and an exposure time of 0.5 s. A total of 180 frames were collected with an oscillation range of 1° and an exposure time of 0.5 s for the MsEgtE-PLP-pyruvate crystal. For the MsEgtE(Y106F) -PLP-geminal diamine crystal, 359 frames were collected with an oscillation step of 1° per frame and an exposure time of 1 s. The distance between the MsEgtE-PLP or MsEgtE-PLP binding pyruvate crystals and the detector is 250 mm, and the distance of the MsEgtE(Y106F) -PLP-geminal diamine crystal and the detector is 150 mm. Diffraction data were processed and scaled with *HKL*-2000 (51). All crystallographic calculations were performed with the CCP4 software package, and the model building and calibration were carried out in Coot (52, 53). A summary of the data collection and processing statistics is shown in Table S1.

To ascertain if the absence of Schiff base bond is a consequence of radiation damage, MsEgtE-PLP dataset was further divided into two parts, frames 1 to 180, and 181 to 361, representing the early and late stages of the diffraction. The structures were refined without any restraint on the linkage between PLP and Lys203.

### PLP content determination

The content of cofactor PLP was determined by adding NaOH to purified enzymes with a final concentration of 0.2 M to denature the enzymes and release the tightly bound PLP. Denatured enzymes were removed by centrifugation at 16,000 rpm for 10 min. The absorbance at 390 nm is measured, and the resulting value was compared with a free PLP quantification standard line to determine the content of PLP (54).

### MsEgtE kinetic characterization

The enzymatic activities of wild-type MsEgtE and mutants were determined using a coupled assay as previously described (55). In a total reaction volume of 200 μl, 10 nM purified enzymes (incubated with PLP) were incubated with 0.13 mM NADH, 1 mM DTT, 22.5 U/ml LDH, and various amounts of sulfoxide 4 in 50 mM KPi buffer, pH 8.0 at 25 °C. The reaction is monitored at 340 nm using SpectraMax iD5. A standard absorbance curve for NADH at 340 nm was used for the calculations of the enzymatic activities. The data were fitted by MATLAB 2022b.

### Determination of the affinity of MsEgtE for sulfoxide four

Isothermal titration calorimetry (ITC) has served as a powerful tool to characterize enzyme kinetics. We adopted the single injection method (SIM) to determine the enzyme kinetics of MsEgtE. All ITC experiments were conducted on a Microcal PEAQ-ITC at 25 °C. 1 mM sulfoxide 4 was loaded in the injection syringe and injected into the sample cell containing 20 μM purified enzymes (one injection 35 μl over 70 s).

### Sequence alignment and phylogenetics

Sequence alignment of MsEgtE from *M. smegmatis* and MtEgtE from *M. tuberculosis* was performed using ClustalW. Phylogenetic tree analysis of EGT-biosynthesis C-S lyases from fungi and bacteria was performed by ClustalW and MEGA-X. The structure-based sequence alignment of EGT-biosynthesis C-S lyases was artificial. The above results were visualized using ESPript3.0 (56).

## Data availability

The atomic structures of MsEgtE-PLP, MsEgtE-PLP binding pyruvate and MsEgtE(Y106F) -PLP-geminal diamine have been deposited in the Protein Data Bank (PDB) under accession numbers 8IRZ, 8IRY and 8IS0 respectively.

## Supporting information

This article contains supporting information.

## Conflict of interest

The authors declare that they have no conflicts of interest with the contents of this article.
