# Supplementary material for: Structure of mycobacterial ergothioneine-biosynthesis C-S lyase EgtE
Source: J Biol Chem. 2023 Dec 10;300(1):105539. doi: 10.1016/j.jbc.2023.105539 (PMC10805701; doi:10.1016/j.jbc.2023.105539)
Supplement: Supporting information [file mmc1.pdf]

Supporting Information

Structure of Mycobacterial Ergothioneine-Biosynthesis C-S lyase EgtE

Lili Wei, Lei Liu*, Weimin Gong*

*Corresponding author. Email: wgong@ustc.edu.cn (Weimin Gong);

lycan@mail.ustc.edu.cn (Lei Liu).

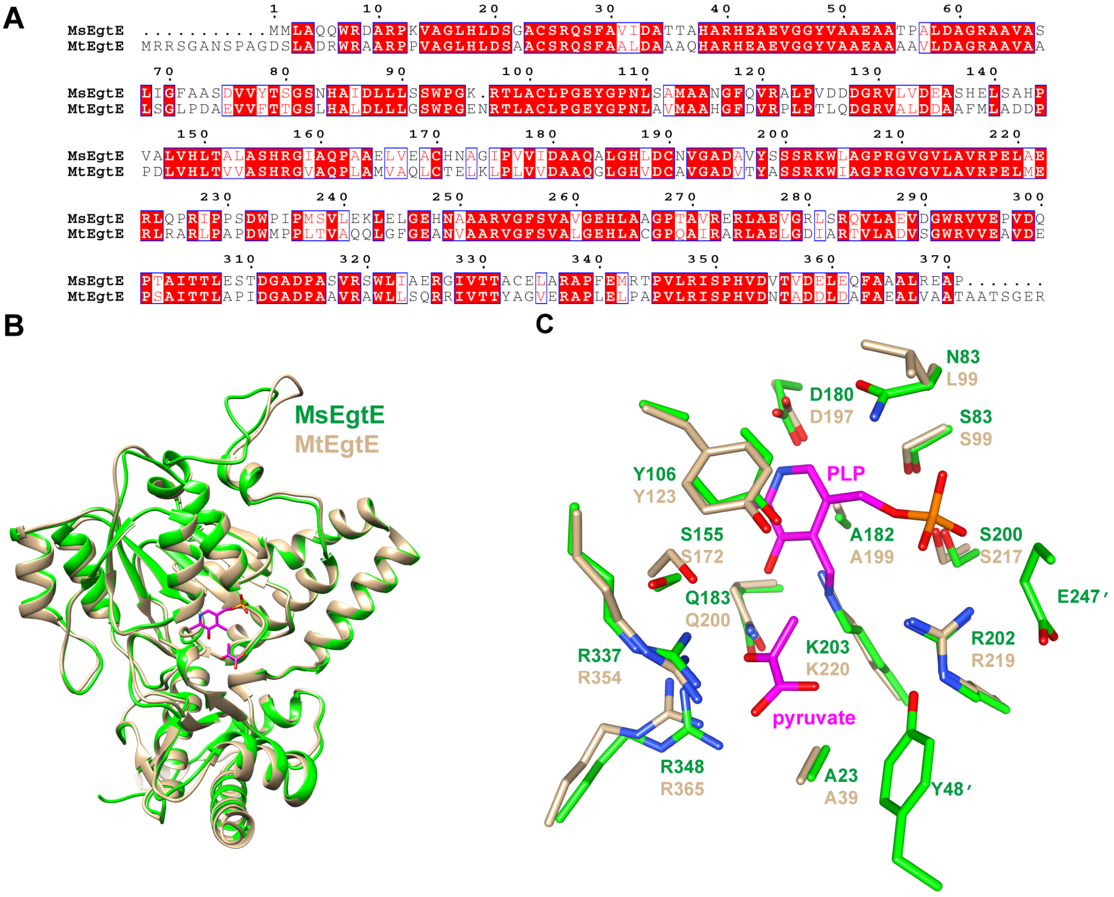

Figure S1. (**A**) Sequence alignment of MsEgtE from *M. smegmatis* and MtEgtE from *M. tuberculosis*. (**B**) Structural alignment of MsEgtE (green, PDB code: 8IRZ) and MtEgtE (tan, AlphaFold code: AF-O69668-F1). **(C)** Comparison of the active center in MsEgtE (green) and MtEgtE (tan), the PLP and pyruvate in MsEgtE are shown as magenta sticks.

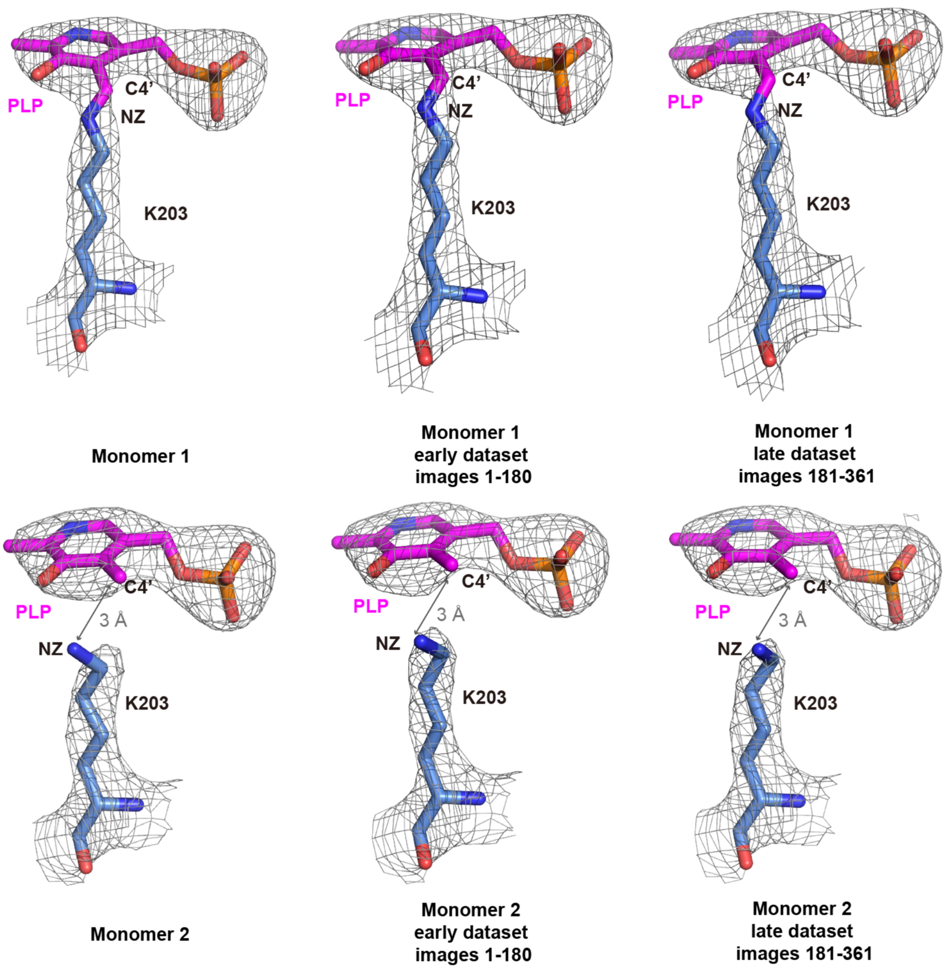

Figure S2. Two states of PLP binding in the active center. The 2Fo-Fc electron density maps of PLP and Lys203 are shown with a gray-colored mesh, and contoured at 1.5σ.

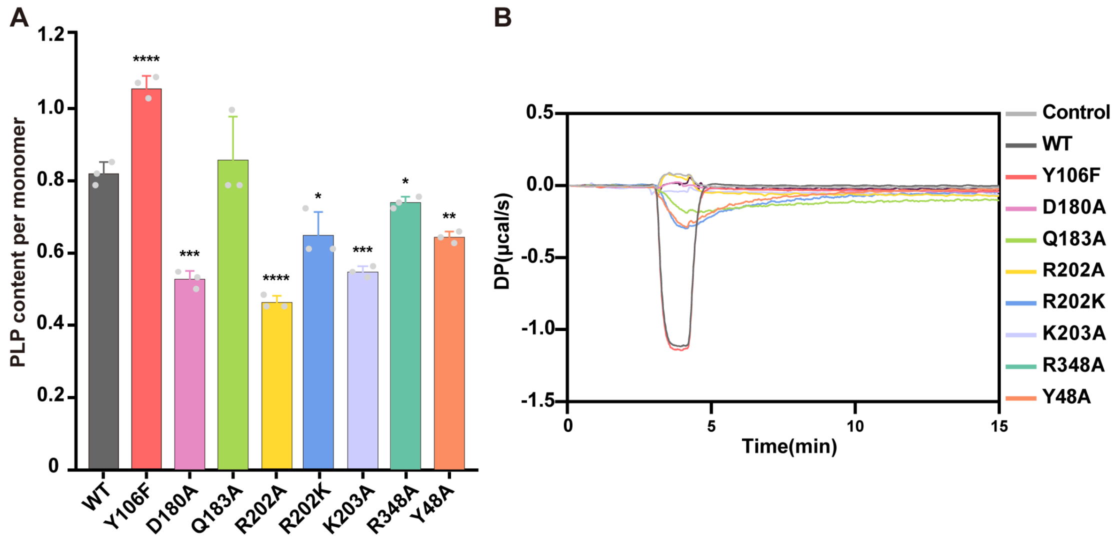

Figure S3. (A) The PLP content per monomer of EgtE and mutants. Results are shown as means ± SD (n = 3). Unpaired t test was used for statistical analysis (*P<0.05, **P<0.01, ***P<0.001, ****P < 0.0001). **(B)** Single injection ITC enzyme kinetic data. Single injection assay of different enzymes (wild type MsEgtE and its mutants) in the sample cell and substrate (sulfoxide 4) in the syringe.

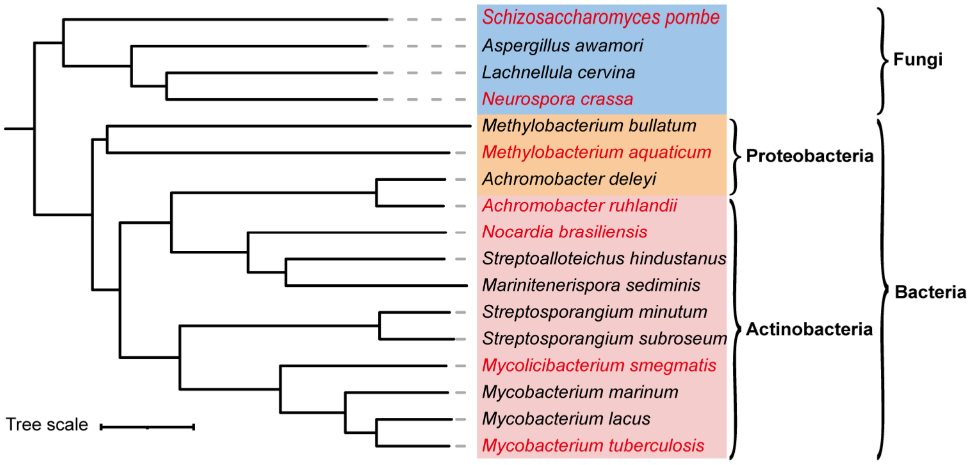

Figure S4. Phylogenetic analysis of EGT-biosynthesis C-S lyases. Amino acid sequences were aligned using ClustalW and a neighbour-joining tree was constructed using the MEGA-X software. Species mentioned in the text are marked red. The consensus phylogenetic tree is shown with bootstrap values >80%.

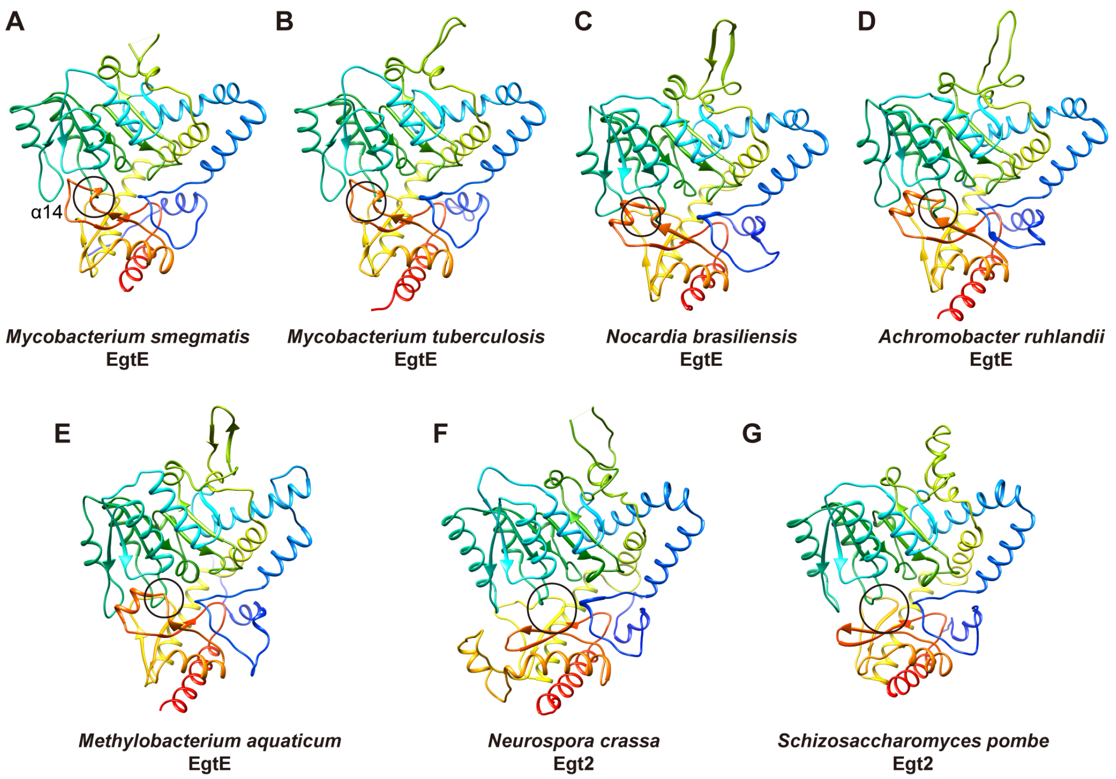

Figure S5. Structural comparison of EGT-biosynthesis C-S lyases. (A) *Mycobacterium smegmatis* EgtE (PDB code: 8IRZ); **(B)** *Mycobacterium tuberculosis* EgtE (AlphaFold code: AF-O69668-F1); **(C)** *Nocardia brasiliensis* EgtE (AlphaFold code: AF-A0A6G9XZ64-F1); **(F)** *Neurospora crassa* Egt2 (PDB code: 5V1X); **(D-E, G)** Predicted structures of EGT-biosynthesis C-S lyases in different species. Significant different regions of these structures are circled with the black line. (Running AlphaFold2 in Google CoLab: AlphaFold2.ipynb - Colaboratory (google.com)).

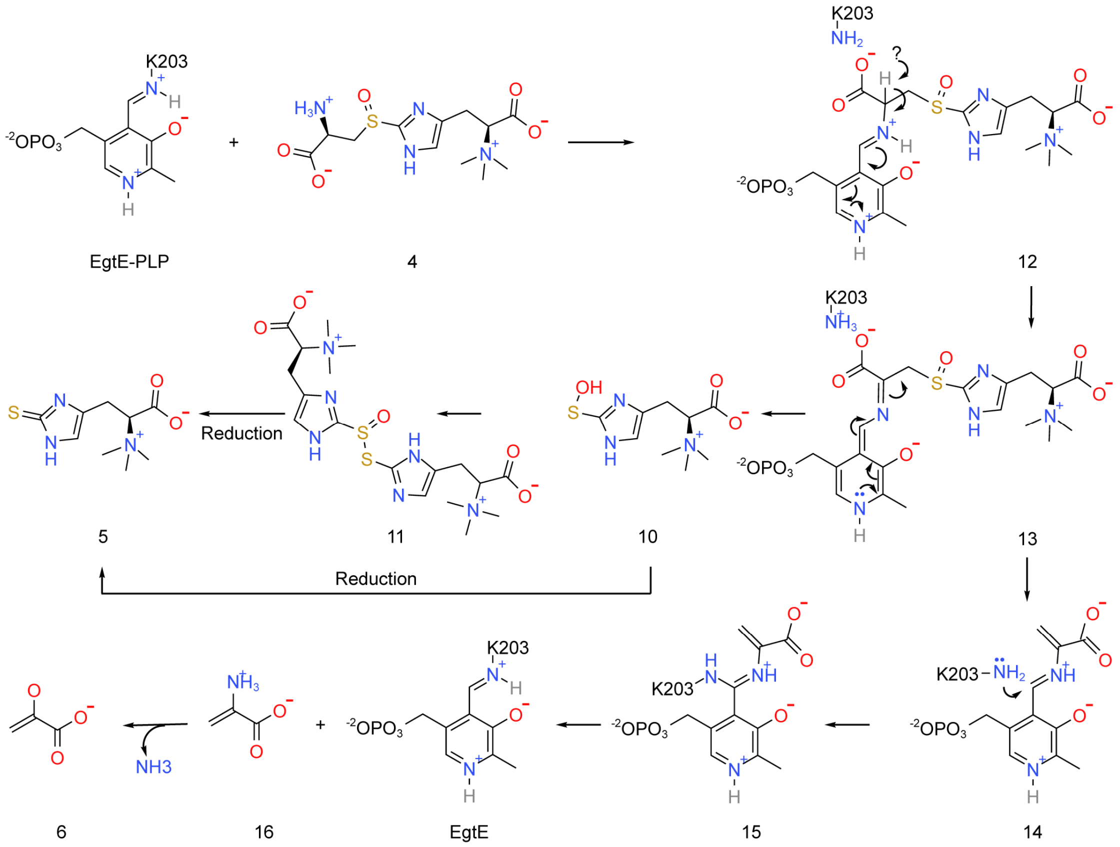

Figure S6. Chemical Reaction Mechanism by EgtE. First, PLP and sulfoxide 4 form an external aldimine intermediate 12, the Schiff base bond between Lys203 and PLP is broken. The Cys α carbon is then deprotonated, leading to the formation of quinonoid intermediate 13. C-S bond cleavage generates ergothioneine sulfenic acid 10 and PLP-based aminoacrylate intermediate 14 which is attacked by the side chain amino group of Lys203 to form intermediate 15. Finally, intermediate 16 is released from EgtE-PLP, followed by imine hydrolysis, resulting in the production of pyruvate and ammonia as by-products. Ergothioneine sulfinic acid 10 is released from the active center. Due to its instability, the disproportionation reaction between the two ergothioneine sulfinic acid 10 molecules will result in the formation of thiol ester of thio-sulfinic acid 11. Alternatively, it can be reduced by thiol reduction system to produce the final product, EGT.

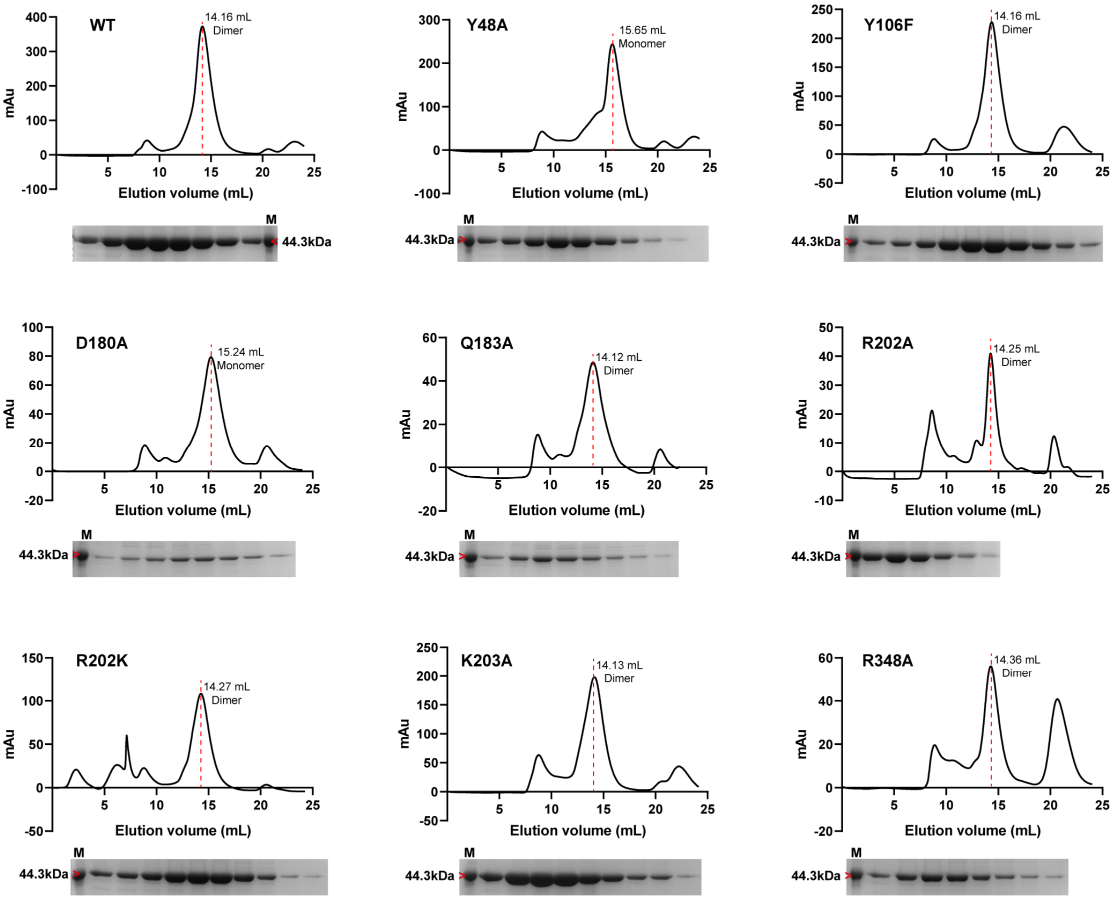

Figure S7. Size-exclusion chromatography elution profiles of WT EgtE and mutants. Lane M, molecular size marker.

Table S1. Data collection and refinement statistics.

**Data collection**	**EgtE-PLP**	**EgtE-PLP binding pyruvate**	**Y106F-PLP-geminal diamine**
Space group	*C121*	*P1*	*C121*
Wavelength (Å)	0.979	0.979	0.979
**Cell dimensions**			
a, *b*, *c* (Å)	108.97, 174.40, 84.09	82.31, 90.37, 101.53	107.52, 173.27, 82.86
α, β, γ (°)	90, 125.82, 90	110.77, 105.95, 103.07	90, 125. 51, 90
Resolution (Å)	50 -2.88 (2.93-2.88)	50-2.34 (2.38-2.34)	50-3.00 (3.05-3.00)
R_{merge}	0.085 (0.545)	0.045 (0.6)	0.186 (1.390)
$I/\sigma I$	12.6 (0.73)	24.7 (1.8)	9.3 (1.2)
Completeness (%)	99.5 (99)	93.4 (92.5)	99.2 (99.9)
Redundancy	4.6 (4.7)	1.6 (1.7)	6.9 (6.9)
**Refinement**			
No. reflections	27573	90294	22555
R_{work} / R_{free}	0.17/0.20	0.16 (0.21)	0.19 (0.24)
No. atoms			
Protein	5497	11064	5524
PLP	30	60	30
Pyruvate	-	6	-
Germinal diamine	-	-	12
Water	21	299	38
B-factors			
Protein	86.97	54.00	83.88
PLP	68.97	38.95	59.26
Pyruvate	-	66.36	-
Germinal diamine	-	-	84.99
Water	63.09	46.78	52.25
R.m.s. deviations			
Bond lengths (Å)	0.003	0.003	0.005
Bond angles (°)	0.941	0.979	1.201
Ramachandran statistics (%)			
Favored	93.43	93.64	91.55
Allowed	5.93	5.49	6.71
Outliers	0.94	0.87	1.74

Highest resolution shell is shown in parenthesis.

Table S2. Primer sequences used in this study.

Name	Sequence (5' to 3' direction)	Usage
MsEgtE-F	GGAATTCCATATGGTGATGCTCGCGCAGCAGTG	Induced expression in *E. coli*
MsEgtE-R	GATCTCGAGTTAGGGCGCCTCACGCAACG	
MsEgtE-Y48A-F	GGCCGAGGTGGGTGGTGCTGTGGCGGCCGAGGCTGC	Mutagenesis
MsEgtE-Y48A-R	GCAGCCTCGGCCGCCACAGCACCACCCACCTCGGCC	
MsEgtE-Y106F-F	CCTGCCTGCCCGGCGAGTTCGGGCCGAATCTGTCTGC	
MsEgtE-Y106F-R	GCAGACAGATTCGGCCCGAACTCGCCGGGCAGGCAGG	
MsEgtE-D180A-F	CCCCGTGGTGATCGCTGCCGCGCAGGCGC	
MsEgtE-D180A-R	GCGCCTGCGCGGCAGCGATCACCACGGGG	
MsEgtE-Q183A-F	GTGATCGACGCCGCGGCGGCGCTGGGGCATCTGG	
MsEgtE-Q183A -R	CCAGATGCCCCAGCGCCGCCGCGGCGTCGATCAC	
MsEgtE-R202A-F	GGTGTACTCATCGTCGGCCAAGTGGCTCGCCGGCCCG	
MsEgtE-R202A -R	CGGGCCGGCGAGCCACTTGGCCGACGATGAGTACACC	
MsEgtE-R202K-F	GCGGTGTACTCATCGTCGAAGAAGTGGCTCGCCGGCCCG	
MsEgtE-R202K -R	CGGGCCGGCGAGCCACTTCTTCGACGATGAGTACACCGC	
MsEgtE-K203A-F	GTACTCATCGTCGCGCGCGTGGCTCGCCGGCCCGCG	
MsEgtE-K203A-R	CGCGGGCCGGCGAGCCACGCGCGCGACGATGAGTAC	
MsEgtE-R348A-F	GCGCACGCCGGTGCTGGCAATCTCGCCGCACGTC	
MsEgtE-R348A-R	GACGTGCGGCGAGATTGCCAGCACCGGCGTGCGC	

The enzyme restriction sites are underlined.

The mutated nucleotide bases are highlighted in red.
